# The Effect of an Enamel Matrix Derivative (Emdogain) Combined with Bone Ceramic on Bone Formation in Mandibular Defects: A Histomorphometric and Immunohistochemical Study in the Canine

**DOI:** 10.1100/2012/196791

**Published:** 2012-04-24

**Authors:** Reza Birang, Mohammad Shah Abouei, Sayed Mohammad Razavi, Peyaman Zia, Ahmad Soolari

**Affiliations:** ^1^Department of Periodontics, School of Dentistry and Torabinejad Research Centre, Isfahan University of Medical Sciences, Isfahan, Iran; ^2^Department of Pathology, School of Dentistry and Torabinejad Research Centre, Isfahan University of Medical Sciences, Isfahan, Iran; ^3^Diplomate of American Board of Periodontology, Private Practice in Periodontics, Silver Spring and Potomac, 11616 Toulone Dr., Potomac, Maryland, MD 20854, USA

## Abstract

*Background*. The purpose of this study was to evaluate the combination of an enamel matrix derivative (EMD) and an osteoconductive bone ceramic (BC) in improving bone regeneration. *Materials and Methods*. Four cylindrical cavities (6 × 6 mm) were prepared bilaterally in the mandible in three dogs. The defects were randomly assigned to four different treatments—filled with EMD/BC and covered with a nonresorbable membrane, filled with EMD/BC without membrane, membrane coverage only, or control (left untreated)—and healed for 2, 4, or 6 weeks. Harvested specimens were prepared for histologic, histomorphometric, and immunohistochemical analyses. *Results*. Sites treated with EMD/BC with or without membrane showed more total bone formation and lamellar bone formation than membrane-only and control defects. There were no statistically significant differences in total bone formation between EMD/BC with or without membrane. *Conclusion*. EMD with BC might improve bone formation in osseous defects more than membrane coverage alone; the use of a membrane had no significant additive effect on total bone formation.

## 1. Introduction

Historically, augmentation or “regeneration” of alveolar bone lost as a result of tooth extraction, periodontal disease, and/or trauma has presented a significant challenge. In this context, many different materials and techniques have been developed and evaluated [1]. Enhancement of bone formation, improvement of the quality of the new bone, shortening of the treatment period (especially for implant treatment), simplified application of materials, and promotion of the effectiveness of biological processes are the primary goals of regenerative treatment approaches [1, 2]. Incorporation of biologic mediators into bone graft materials or other carriers in bone augmentation process and regeneration of bone defects around the tooth or/and implant may help improve outcomes [1, 3].

Emdogain is a mixture of enamel matrix derivatives (EMDs) that can be used as an osteopromotive agent for the aforementioned bone augmentation/regeneration treatments [1]. Emdogain has been shown to enhance the osteogenic potential of bone marrow by increasing the total number of stromal cells [4–6], enhancing the proliferation of osteoblasts [7–12], promoting cell differentiation [7, 13–16], and stimulating migration and viability of osteoblasts [12], which can lead to improved bone regeneration. Thus, in addition to using EMDs for treatment of periodontal lesions [17–19], the application of EMDs to improve bone healing and increase the rate of bone formation has been proposed [19–23]. However, because of its gelatinous nature, Emdogain is not effective in preserving the space for new bone formation [23]; in addition, it has only “osteopromotive” properties, and its osteoinductive properties have not yet been proven [24–27]. These observations have spurred considerable research interest in the incorporation of EMDs into a variety of bone replacement grafts to enhance wound stability and maintain space [26–34], but controversy remains regarding the results of these combination therapies.

Bioactive ceramics such as hydroxyapatite (HA), tricalcium phosphate (TCP), or a combination of them (into biphasic calcium phosphate) are biocompatible materials that provoke no or little inflammatory response and have been accepted as bioactive osteoconductive scaffolds for new bone formation and the ingrowth of osteoprogenitor cells [35, 36]. Biphasic calcium phosphate biomaterials have been developed to achieve a better result in living tissues than pure TCP or HA alone [30, 31, 37, 38]. Also, it has been shown that the same amount of new bone is achieved but with less residual graft material comparing with other bone graft materials [39]. Biphasic calcium phosphate, a bone ceramic (BC) with slow dissolution and substitution rates as well as osteoconductive properties, could possibly act as a carrier for bone-stimulating proteins [36].

Therefore, the aim of this study is to propose a new method for improving bone regeneration using a combination of Emdogain and osteoconductive BC.

## 2. Method and Materials

Three male canines of a mixed Iranian breed and nearly identical weights (25 kg) were selected for the study. Animal selection, management, and surgical protocols were accomplished in accordance with the guidelines of the animal and human experimentation committee of Isfahan University of Medical Sciences.

### 2.1. Surgical Procedure

General anesthesia was induced by injecting acepromazine 1% (Neurotranq, Alfasan, Woerden, The Netherlands; 0.02 mL/kg) and ketamine hydrochloride 10% (Ketamine, Alfasan; 0.04 mg/kg) and was completed with inhalation anesthetic (Halothane BP, Nicholas Piramal India Limited, India). The oral area was rinsed with chlorhexidine 0.2% and then placed under local anesthesia (Persocaine E, Lidocaine HCl 2% + epinephrine 1/80,000; Daru Pakhsh Pharmaceutical Mfg Co, Tehran, Iran). All mandibular premolars were then extracted.

**Figure 1:**
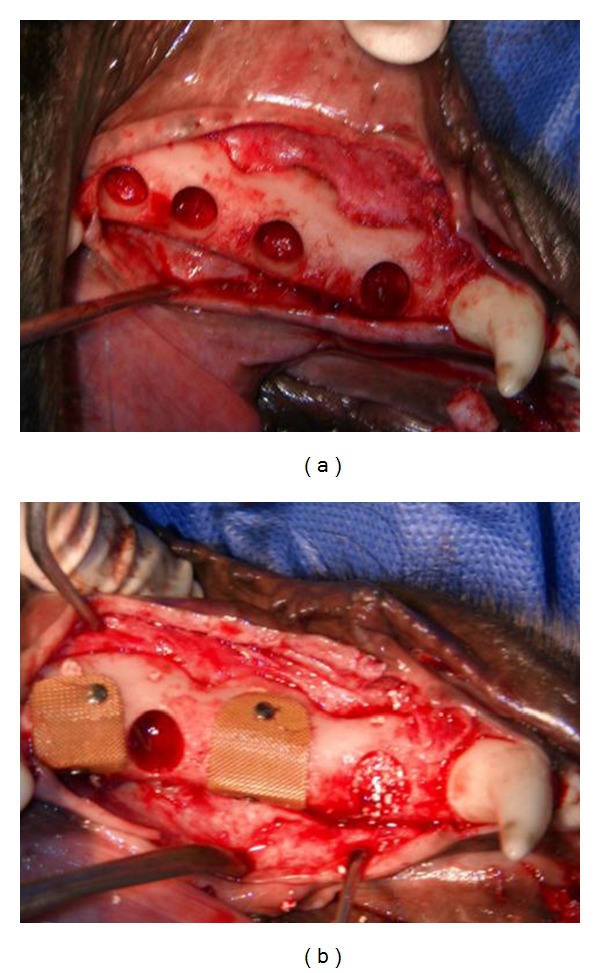
(a) Four cylindrical defects, 6 mm deep and 6 mm in diameter, were prepared on each side of the mandible. (b) The four types of treated defects.

Postoperative antibiotics, including penicillin and streptomycin (Nasr Pharmaceutical Co, Fariman, Iran; 40,000 IU/kg), were prescribed for 7 days, and tramadol (Tehran Chemic Pharmaceutical Co, Tehran, Iran; 5 mg/kg) was applied for pain control. After 3 months of healing, the animals were anesthetized as before; then, under local anesthesia, full-thickness flaps were created bilaterally by making a crestal incision on the mandibular alveolar processes 3 mm from the canines to 2 mm from the first molars. With a trephine bur (No. 6, Meisinger, Neuss, Germany), four standardized cylindrical cavities with a depth and diameter of 6 mm were created on each side of the mandible (3 mm from each other) (Figure 1). Also, small holes were drilled on the apical and lateral aspects of each cavity with a round No. 2 bur that was filled with gutta-percha. The bone defects on each side were randomly assigned to one of the following four groups (Figure 1).

- EMD/BC with membrane: the defect was filled with a combination of Emdogain (Institut Straumann, Basel, Switzerland) and BC (OSTEON, Geonosis, Suwan-si, Korea) and covered with a nonresorbable membrane (Osteo-Mesh TM-300, CYTOPLAST, Osteogenic Biomedical Inc, Lubbock, TX, USA) that was fixed with tacks (FRIOS, Dentsply/Friadent, Mannheim, Germany).
- EMD/BC without membrane: the defect was filled with a combination of Emdogain and BC.
- Membrane only: the defect was covered only with a nonresorbable membrane that was held in place with tacks.
- Control: the defect was left untreated.

The flaps were repositioned and closed with resorbable polyglycolic acid sutures (PGA, TEB Keyhan, Eshtehard, Iran). At the end of surgery, all animals received intramuscular injections of antibiotics and analgesics, as with the first surgery. During the first 2 weeks after the surgery, the dogs' mouths were washed with chlorhexidine 0.2%, and the animals were fed a soft diet rich in supplementary vitamins.

### 2.2. Retrieval of Specimens

The animals were checked twice per day during the first postoperative week for signs of infection. The three animals were randomly assigned to three different time intervals: 2 weeks, 4 weeks, and 6 weeks; at the end of each designated healing period, each animal received general anesthesia and was sacrificed by vital perfusion.

Each mandible was sectioned and fixed in 10% neutral buffered formalin. Eight harvested blocks containing the specimens were obtained from each mandible (four specimens from each side, one from each group). They were decalcified in 10% ethylenediaminetetraacetic acid for 8 weeks and then embedded in acrylic resin (Meliodent, Heraeus Kulzer, Newbury, Berkshire, United Kingdom). With a microtome (ACCU-Cut SRM200, Sakura Finetek Europe, Alphen aan den Rijn, The Netherlands), buccolingual cross-sections were obtained from the middle portion of the defects and were ground to a final thickness of 4 *μ*m. Two slides of each specimen were stained with hematoxylin-eosin, and the other two slides were prepared for immunohistochemical analysis for markers of osteopontin (OPN) (Ncl-o-pontin, mouse monoclonal, Leica Biosystems, Newcastle, United Kingdom) by the polymer HRP method according to the manufacturer.

### 2.3. Histologic and Histomorphometric Analyses

Slides were examined by a blinded examiner under a light microscope (Olympus CX21FS, Olympus Corporation, Tokyo, Japan) at a magnification of ×100. For each section, the percentages of total generated bone, woven bone, lamellar bone, and existing fibrous connective tissue were measured.

### 2.4. Immunohistochemical Analysis

The intensity of OPN expression in bone matrix was examined on each slide by light microscope. Based on the observation, OPN staining intensity was ranked as no expression (−), mild expression (+), moderate expression (++), or strong expression (+++).

## 3. Results

In this study, membrane exposures were observed in some surgical sites during healing. In these cases, the membrane was removed at 4 weeks, and the site was resutured in the canine that healed for 6 weeks.

**Figure 2:**
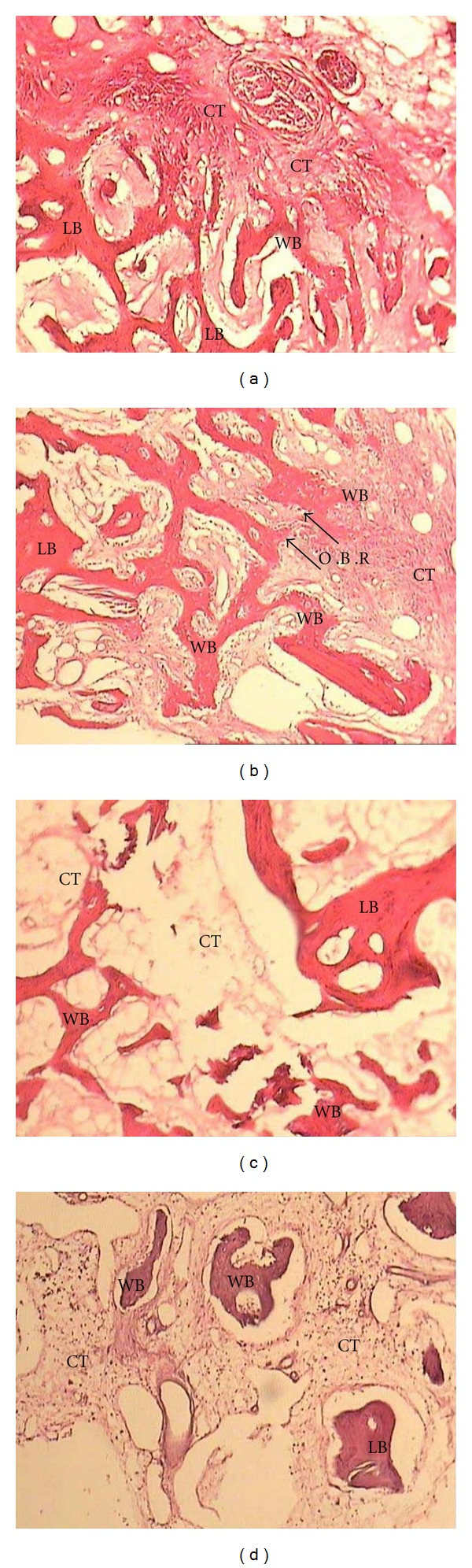
Histological views of new bone formation with hematoxylin-eosin staining (magnification ×100). (a) GBR group; (b) EMD/BC without membrane; (c) EMD/BC with membrane; (d) control group. CT: connective tissue, LB: lamellar bone, WB: woven bone, OBR: osteoblastic rim.

Histologic examination revealed that new bone had formed in all experimental groups, especially in the apical portions of the defects. Acute inflammation was observed in only one specimen (from a site treated with EMD/BC with membrane at 2 weeks) (Figure 2). The presence of both lamellar and woven bone in the specimens at all intervals (2, 4, and 6 weeks) suggested that bone remodeling was taking place. Also, some residual materials were found in some specimens in the EMD/BC-treated sites.

**Figure 3:**
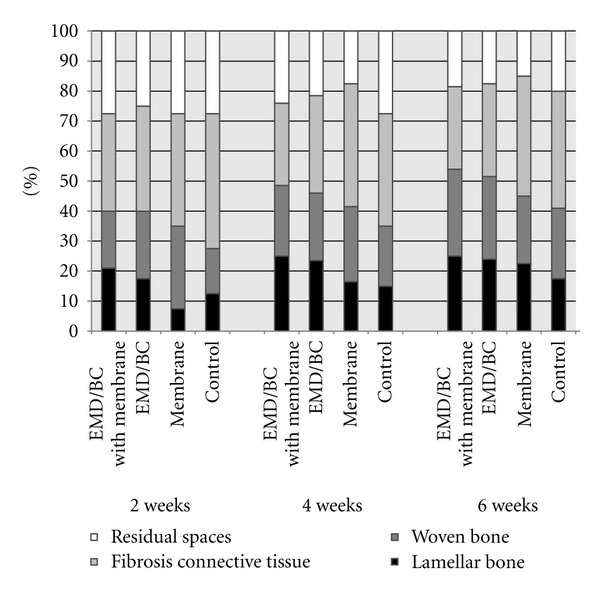
The mean percentages of lamellar bone, woven bone, and fibrous connective tissue for each experimental group and time interval. EMD/BC + M: EMD/BC with membrane; EMD/BC: EMD/BC without membrane; M: membrane only; C: control.

Table 1 and Figure 3 present the results of histomorphometric evaluation of the different groups and intervals. Repeated-measures analysis of variance showed that there were statistically significant differences in formation of total bone, lamellar bone, and woven bone as well as the in the existing fibrous connective tissue between the groups (*P* < .05). Based on the paired *t*-test, both EMD/BC with and without membrane showed statistically significant differences in total bone formation and lamellar bone formation compared with the membrane-only and the control groups (*P* < .05). However, there was a statistically significant difference in woven bone formation only with the control group. EMD/BC with membrane had the most lamellar bone formation, but this was not statistically significantly different versus EMD/BC without membrane in the mean percentages of total and woven bone formation (paired *t*-test, *P* > .05). Also, both EMD/BC experimental groups (both with and without membrane) showed a statistically significant difference in existing fibrous connective tissue with the membrane-only and control groups, as well as with each other (*P* < .05).

**Figure 4:**
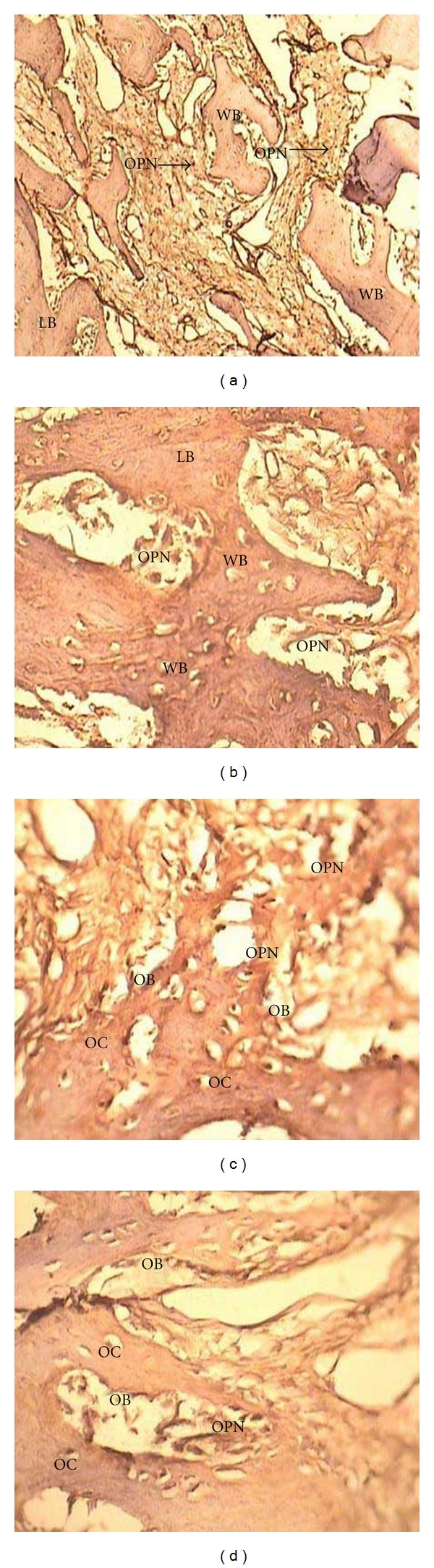
Immunohistochemical views of OPN stain intensity. ((a), magnification ×100) EMD/BC with membrane; ((b), ×400) EMD/BC without membrane; ((c); ×400) GBR group; ((d), ×400) control group. LB: lamellar bone, WB: woven bone, OB: osteoblast, OC: osteoclast, OPN: osteopontin-expressing cell.

The results of immunohistochemical evaluation and the intensity of staining for OPN in the different groups are shown in Table 2; samples are shown in Figure 4. The Friedman test demonstrated that there were statistically significant differences between all experimental groups, so the Wilcoxon test was used to compare individual pairs of groups, with the level of significance set at *α* = .05. It revealed that only EMD/BC with membrane showed a statistically significant difference versus the membrane-only and control groups. There were no statistically significant differences between the other experimental groups regarding the intensity of staining for OPN.

## 4. Discussion

In the present study, in the defects treated with EMD/BC (with or without membrane), the mean percentage of new bone formation was greater than that seen the control and membrane-only groups during each interval. Also, the quality of the newly formed bone was improved, as evidenced by the higher percentage of lamellar bone formed in the EMD/BC groups. This improvement in bone formation might have resulted from the combined effects of EMD and BC. Emdogain is a commercially available mixture of EMDs. The composition of EMDs has been described as a hydrophobic enamel matrix protein complex derived from 6-month-old porcine tooth buds containing more than 90% amelogenin as well as enamelin, tuftelin, tuft proteins, ameloblastin [40], and other peptides such as bone morphogenetic proteins (BMPs) and transforming growth factor-beta (TGF-*β*) [41, 42]. It has been known that amelogenins are self-assembled into supermolecular aggregates, which are generated in insoluble extracellular matrix [43] with high affinity for collagens and HA [44, 45].

In vitro studies have demonstrated that EMDs stimulate bone cell proliferation and differentiation [5, 7, 8, 11, 12, 30, 46], affecting bone formation. Takayama et al. reported that BMP-like molecules (BMP-2, BMP-4, and BMP-7) in EMDs encourage the promotion of osteogenic differentiation [14]. Another study reported that the presence of EMDs can inhibit myoblastic development of cultured pluripotential mesenchymal cells and increase alkaline phosphatase activity [13]. Goda et al. reported that the BMP-2 and TGF-*β* in Emdogain can activate osteoblasts and enhance the production of collagenase (i.e., matrix metalloproteinase-1), which degrades matrix proteins in bone tissue microenvironments, resulting in the facilitation of bone regeneration [47, 48].

On the other hand, the HA/TCP carrier acts as a scaffold [36, 49]. This scaffold can play an important role in facilitating the attachment of stimulated cells, as well as promoting these cells to produce new bone [36]. It has been shown that biphasic calcium phosphate can increase the concentration of free calcium ions in the environment, which acts as a calcium reservoir, thereby assisting with bone formation [50].

The increased bone formation shown in the present study is in agreement with other in vivo investigations that have demonstrated the role of enamel matrix proteins in the regeneration of dehiscence type defects around implants [21] and its osteopromotive effect on bone regeneration during the healing of injured bones [20]. Also, some studies have shown that the use of Emdogain in combination with bone graft materials can improve bone formation [29–34]. Potijanyakul et al. demonstrated that rat calvarial defects filled with bioactive glass plus EMD showed a slightly higher percentage of new bone formation than those filled with bioactive glass alone [31]. In another study, the effect of a mixture of EMD and *β*-TCP on bone augmentation within a titanium cap in rabbit calvaria was evaluated. It was shown that the EMD promoted initial bone formation and maturation of mineralized bone after 1 month. However, there was no significant difference in the amount of newly formed bone in EMD plus *β*-TCP compared with *β*-TCP alone in the 3-month follow-up group. Hence, the authors suggested that EMD did not promote osteoblastic activity but encouraged osteogenic differentiation of pluripotent mesenchymal cells [24]. Another study demonstrated that, although the application of Emdogain with a membrane resulted in a slight (but not significant) increase in vertical bone formation, the addition of Emdogain to bone graft materials did not have any additional benefits [23]. The results of previous studies regarding the role of EMDs in bone formation have proposed that Emdogain was more effective when it was combined with bone substitute materials for the treatment of periodontal osseous defects [32, 51–53]. In contrast, other studies have found that Emdogain in combination with bone substitute did not significantly enhance the potential for total bone formation in osseous defects [27, 30, 54].

The difference in bone formation and osteogenic potential of EMD with or without bone grafting materials can be explained either by the different EMD concentrations that were used or by the bone inductive properties of specific bone substitute materials. Additionally, it is worth mentioning that if Emdogain does not increase bone formation or osteoinduction, the possibility that EMDs are involved in osteoblastic differentiation in general is not necessarily excluded. Also, in vitro and in vivo studies have documented that amelogenin proteins have osteogenic potential [23, 45, 55]. Thus, it could be concluded that the Emdogain used in different studies might not contain the appropriate amount of amelogenins with osteogenic potential and has lower concentrations of EMDs and amelogenin than is necessary for achieving bone formation and osteoinduction.

Application of a membrane along with EMD/BC in the present study resulted in a decrease in the existence of fibrous connective tissue and an increase in lamellar bone formation. However, it did not have any significant additional effect on total bone formation or woven bone formation, which is consistent with some previous studies [23, 32].

In the present research, immunohistochemical evaluation showed that the defects treated with a combination of Emdogain and BC (with or without a membrane) had the most intense staining, indicating more extracellular OPN expression in these defects in comparison with the other treatments. OPN is a noncollagenous phosphorylated acidic glycoprotein that resides in the extracellular matrix of mineralized tissues and is produced by osteoblasts, osteoclasts, osteocytes, preosteoblasts, some bone marrow cells, and many nonbone cells [56, 57]. It has been shown that OPN can bond to HA and calcium ions with its arginine-glycine-aspartate sequence [57]. OPN acts as an important factor in bone remodeling, wound repair, angiogenesis, cell survival, immune function, and several pathophysiological processes [56, 58]. In mineralized tissue, OPN is secreted by both osteoblasts and osteoclasts, and its concentration in areas of newly formed bone should be increased [59]. Some previous immunohistochemical studies have reported a progressive increase, either in OPN detected in maturing membranous bone matrix or in OPN expression by preosteoblasts and osteoblasts in developing mandibular bone [59].

The increased expression of OPN in defects treated by Emdogain/BC suggests that osteoblast differentiation and osteoclastic activity were enhanced, indicating more bone remodeling. Also, according to previous studies, EMDs may accelerate expression of OPN [60], confirming the useful effects of EMDs on periodontal and bone regeneration. While the greatest amount of lamellar bone formation was observed in the defects treated with EMD/BC and a membrane, OPN staining was also intense in this group, confirming its superior bone remodeling. However, there was no significant difference in OPN stain intensity between the sites treated with EMD/BC with or without a membrane. OPN is the important interfibrillar portion of type I collagen in the extracellular matrix of woven bones [61]. Therefore, it is suggested that the lack of significant difference in woven bone formation between these groups is paralleled by the absence of significant differences in OPN stain intensity.

It can be seen as a limitation of this study that BC alone, without EMD, was not tested. However, the primary purpose of the present study was to evaluate the role of a BC in bone regeneration. Historically, bone graft materials have been evaluated with and without a membrane. Future studies will investigate the role of BC in regeneration procedures.

## 5. Conclusion

According to the results, Emdogain combined with bone ceramic (TCP/HA) might improve bone formation in osseous defects more than the use of membrane alone. It was also observed that the use of a membrane in combination with Emdogain and bone ceramic did not confer an advantage with regard to total bone formation.

## Figures and Tables

**Table 1:** Mean percentage (± SDs) of tissue areas for each experimental group.

Experimental group		Tissue type			
	*n*	Total bone formation	Lamellar bone formation	Woven bone formation	Fibrous connective tissue
EMD/BC with membrane	6	47.5 ± 7.55^a^	23.6 ± 2.16^a^	23.8 ± 5.81^a^	29.1 ± 4.44^a^
EMD/BC without membrane	6	45.8 ± 6.14^a^	21.6 ± 3.77^b^	24.1 ± 3.43^a^	32.8 ± 2.48^b^
Membrane only	6	40.5 ± 8.45^b^	15.5 ± 8.45^c^	25.0 ± 3.16^a,b^	39.5 ± 5.04^c^
Control	6	34.5 ± 8.33^c^	15.0 ± 4.47^c^	19.5 ± 5.04^b^	40.5 ± 5.61^c^

^a,b,c^Different superscript letters indicate statistically significant differences (*P* < .05). EMD/BC = Combination of Emdogain with BC (biphasic calcium phosphate, i.e., TCP + HA).

**Table 2:** OPN staining intensity in each experimental group.

Treatment group	Staining intensity	Interval			Total
		2 weeks	4 weeks	6 weeks	
EMD/BC with membrane	−	0	0	0	0
	+	0	0	0	0
	++	1	2	1	4
	+++	1	0	1	2

EMD/BC without membrane	−	0	0	0	0
	+	1	1	1	3
	++	0	1	0	1
	+++	1	0	1	2

Membrane only	−	0	0	1	1
	+	1	1	0	2
	++	1	1	1	3
	+++	0	0	0	0

Control	−	0	0	0	0
	+	2	2	2	6
	++	0	0	0	0
	+++	0	0	0	0

Ratings for OPN expression: −: no expression, +: mild expression, ++: moderate expression, ++++: strong expression.
